# Supplementary material for: Splice-Junction-Based Mapping of Alternative Isoforms in the Human Proteome
Source: Cell Rep. Author manuscript; Available in PMC 2020 Jan 15. (PMC6961840; doi:10.1016/j.celrep.2019.11.026)
Supplement: 2 [file NIHMS1546469-supplement-2.zip › DF1/PXD009737/TestisTrypsin/TestisTrypsin_7_MYCBP2_PNLPAPSIFDQAAKPPSSLVHSPFVFGQPLSFQQPQLQSDR.pdf]

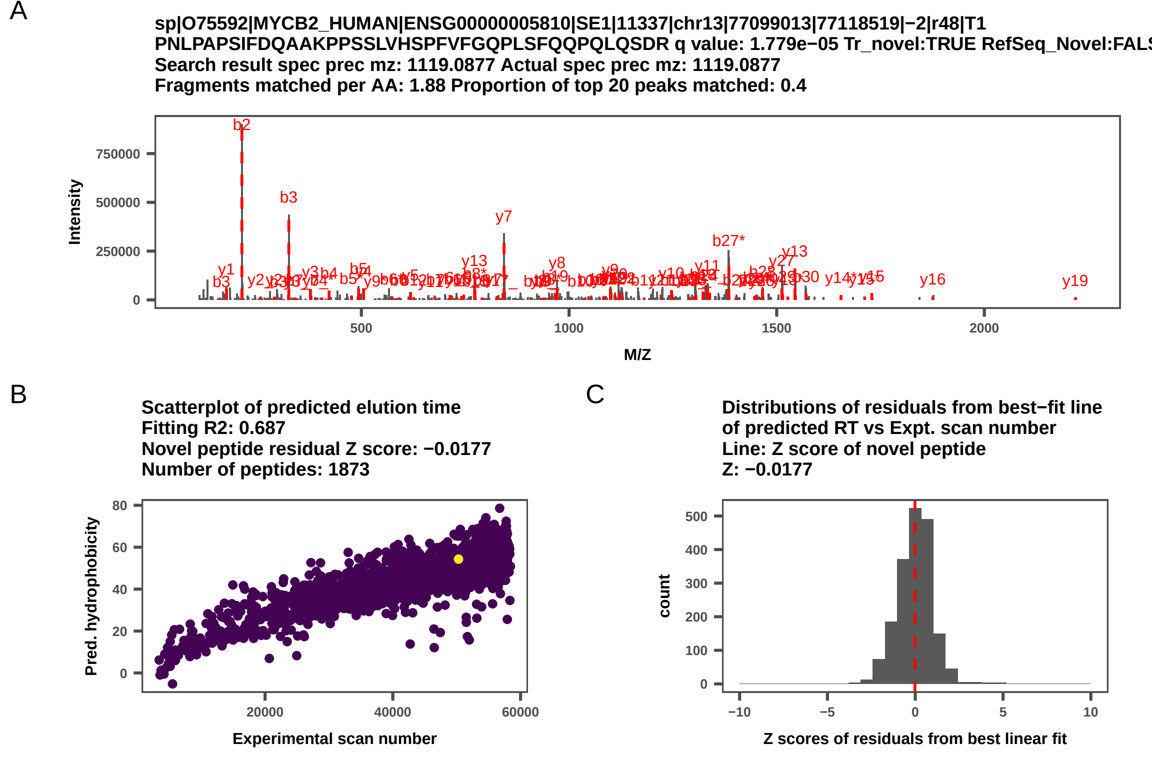
A
sp|O75592|MYCB2_HUMAN|ENSG00000005810|SE1|11337|chr13|77099013|77118519|−2|r48|T1
PNLPAPSIFDQAAKPPSSLVHSPFVFGQPLSFQQPQLQSDR q value: 1.779e−05 Tr_novel:TRUE RefSeq_Novel:FALS
Search result spec prec mz: 1119.0877 Actual spec prec mz: 1119.0877
Fragments matched per AA: 1.88 Proportion of top 20 peaks matched: 0.4
Intensity
750000
500000
250000
0
b2
b3
y7
b27*
y13
y27
y8
y11
y1
y16
y19
500
1000
1500
2000
M/Z
B
Scatterplot of predicted elution time
Fitting R2: 0.687
Novel peptide residual Z score: −0.0177
Number of peptides: 1873
Pred. hydrophobicity
80
60
40
20
0
20000
40000
60000
Experimental scan number
C
Distributions of residuals from best−fit line
of predicted RT vs Expt. scan number
Line: Z score of novel peptide
Z: −0.0177
count
500
400
300
200
100
0
−10
−5
0
5
10
Z scores of residuals from best linear fit
